# Exploring factors influencing delayed first antibiotic treatment for suspected early-onset sepsis in preterm newborns: a study before quality improvement initiative

**DOI:** 10.1186/s12887-024-04887-9

**Published:** 2024-06-26

**Authors:** Jun Chen, Xiaoling Fang, Weidong Liu, Chaomei Huang, Yiheng Dai

**Affiliations:** 1 Division of Neonatology, Foshan Women and Children Hospital, , No. 20, Huayang Road, Foshan, GD 528000 China; 2 Division of Neonatology, Zhujiang Hospital of Southern Medical University, Foshan, Guangdong China

**Keywords:** Neonatal intensive care unit, Preterm infant, Early-onset sepsis, Delayed antibiotic administration, Linear regression analysis

## Abstract

**Background:**

Early-onset sepsis (EOS) is a serious illness that affects preterm newborns, and delayed antibiotic initiation may increase the risk of adverse outcomes.

**Purpose:**

The objective of this study was to examine the present time of antibiotic administration in preterm infants with suspected EOS and the factors that contribute to delayed antibiotic initiation.

**Methods:**

In this retrospective study in China, a total of 82 early preterm infants with suspected EOS between December 2021 and March 2023 were included. The study utilized a linear regression analytical approach to identify independent factors that contribute to delayed antibiotic administration.

**Results:**

The mean gestational age and birth weight of the study population were 29.1 ± 1.4 weeks and 1265.7 ± 176.8 g, respectively. The median time of initial antibiotic administration was 3.8 (3.1-5.0) hours. Linear regression revealed that severe respiratory distress syndrome (RDS) (β = 0.07, *P* = 0.013), penicillin skin test (PST) timing (β = 0.06, *P* < 0.001) and medical order timing (β = 0.04, *P* = 0.017) were significantly associated with the initial timing of antibiotic administration.

**Conclusions:**

There is an evident delay in antibiotic administration in preterm infants with suspected EOS in our unit. Severe RDS, PST postponement and delayed medical orders were found to be associated with the delayed use of antibiotics, which will be helpful for quality improvement efforts in the neonatal intensive care unit (NICU).

## Background

Early-onset sepsis (EOS) is a serious illness that affects preterm newborns and can lead to significant morbidity and mortality if not treated promptly [1]. Empiric antibiotic treatment is often initiated in newborns at risk for EOS while awaiting culture results [2, 3]. However, several studies have suggested that delayed antibiotic initiation may increase the risk of adverse outcomes, including death and neurodevelopmental impairment [1, 4]. For instance, in a retrospective study of 817 infants with sepsis, those for whom antibiotic initiation was delayed (1 h after the onset of clinical signs) had a higher mortality rate than those who received antibiotics within the first hour (14% vs. 6%, respectively) [3].

To enhance the quality of care and optimize patient outcomes, it is essential to identify the factors contributing to the delay in initiating antibiotic treatment in preterm infants with EOS. Previous quality improvement (QI) studies have explored various factors that can be broadly categorized as patient-related, healthcare system-related, and clinician-related factors [5, 6]. However, in this study, we addressed unique challenges specific to our medical unit in China, such as restrictions on gentamicin use and the requirement of a penicillin skin test (PST) before providing penicillin therapy, which may have an impact on the timing of treatment initiation.

In our single-center study, we aimed to comprehensively analyze the timing of antibiotic administration in preterm newborns with EOS. By identifying the factors associated with delayed initiation, we seek to develop effective strategies to enhance prompt antibiotic administration for this vulnerable patient population. The findings will inform targeted interventions in future QI projects to reduce delays and improve the care provided to these infants in our medical center.

## Methods

### Study design

As a component of a QI initiative at our hospital, we conducted a retrospective study to identify the factors associated with the delayed administration of antibiotics in preterm infants with suspected EOS. The study was conducted at a tertiary-level neonatal intensive care unit (NICU) in southern China over a period of 18 months. Importantly, the hospital was relocated to a new area in December 2021, leading to significant changes in workflow. Due to this relocation, data collection for the study was restricted to the period from December 2021 to March 2023.

### Patient selection

The protocol was approved by the Institutional Research Ethics Board (IREB). The study’s inclusion criteria were as follows: infants who were born at a gestational age of less than 32 weeks and presented with symptoms indicative of EOS due to intrapartum infection. The criteria for EOS in this study were from the expert consensus on the diagnosis and management of neonatal sepsis in China (2019) [7]. EOS risk factors include maternal fever during labor, prolonged rupture of membranes (> 18 h), chorioamnionitis, and a positive group B streptococcus (GBS) culture. Clinical signs and symptoms in newborns, such as respiratory distress, apnea, temperature instability, lethargy, poor feeding, and hypotension, may also suggest EOS. Additionally, laboratory findings such as an elevated white blood cell count, a low platelet count, or an abnormal C-reactive protein (CRP) level in mothers may support the diagnosis of EOS. In our center, infants born to mothers with these risk factors are considered at higher risk for EOS and may require evaluation and antibiotic treatment.

### Study setting

In the NICU setting in China, as part of our hospital’s protocol, healthcare providers are required to perform a PST and observe the reaction for a minimum of 20 min before administering penicillin-based antibiotics to preterm infants. While the PST helps minimize the risk of adverse reactions, it can also lead to delays in antibiotic administration [8]. Furthermore, the use of gentamicin in newborns is restricted in China due to the potential risk of irreversible hearing loss and kidney damage in these vulnerable patients. As a result, current guidelines for newborn sepsis recommend empiric antibiotic therapy consisting of third-generation cephalosporins along with penicillin for suspected EOS. Furthermore, the administration of cephalosporins typically involves a continuous infusion over 30 to 60 min, which may also contribute to the delay in antibiotic administration.

### Data collection

To ensure the validity of suspected EOS diagnosis in early preterm newborns, two NICU experts (the first and corresponding authors) independently assessed each case. In cases where a consensus was not reached, the entire team engaged in discussions to decide whether the patient should be included in the study. Demographic and clinical characteristic data were extracted from electronic medical records based on patient identification. The clinical data included sex, gestational age, mode of delivery, ventilation support mode, reintubation and clinical signs of shock.

The electronic nursing system is routinely utilized in our NICU to document the timing of key nursing activities such as patient transfer to the unit, the prescription of antibiotics, performance of a PST, and the administration of antibiotics. Our primary outcome was the time to antibiotic administration, defined as the interval between all prescribed antibiotic administrations and birth. Furthermore, the time of NICU bed allocation was calculated from the newborn’s birth and recorded in the medical system. Similarly, the timing of PST performance was measured as the duration between test administration and the medical order time. The time between the resident doctor’s order and the patient’s appearance in the system was also recorded as the timing of the antibiotic order.

### Statistical analysis

In this study, SPSS Statistics 26.0 software (IBM Corp) was used to process all data. Categorical data are described using percentages and proportions. For continuous variables, such as gestational age and birth weight, that conformed to a normal distribution, the mean ± standard deviation are reported. For variables displaying a nonnormal distribution concerning the timing of antibiotic administration, we report the median and interquartile range (IQR).

Due to the nonnormal distribution of the time variables, a logarithmic transformation was applied. This transformation is commonly employed in statistical analysis to improve normality and meet the assumptions of parametric tests. To assess relationships between the variables, the t test was employed during the univariable analysis to compare time differences across different groups. Additionally, the Pearson correlation coefficient was used to establish associations between the time variables. To identify independent risk factors, potential influencing variables were initially analyzed in a univariate model. Subsequently, only the variables that showed significance at the *P* < 0.1 level were selected for inclusion in the multivariable model. Linear regression was then used to estimate the effect of each predictor on the outcome across different levels of the outcome variable.

## Results

### Participant characteristics

The study included a total of 82 preterm infants with suspected EOS. The mean gestational age and birth weight were 29.1 ± 1.4 weeks and 1265.7 ± 176.8 g, respectively. Of the included patients, 68.3% (*n* = 56) were male, with 75.6% (*n* = 62) being singletons and 24.4% (*n* = 20) being twins. Regarding delivery method, 36.6% (*n* = 30) of the preterm infants were born via emergency cesarean section. Additionally, 31.7% (*n* = 26) of the infants were delivered vaginally, while an equal number of infants were born via elective cesarean Sect. (31.7%, *n* = 26).

### Time-related variables

The median (IQR) duration from delivery to NICU bed allocation was 0.5 (0.4–0.6) hours. The median time from NICU admission to the completion of the antibiotic order was 1.0 (0.6–1.8) hour. The overall median time from birth to the initial administration of antibiotics was 3.8 (3.1-5.0) hours.

### Univariate factor analysis

This study analyzed multiple factors, including multiple pregnancy, gestational age, and sex, to determine their potential associations with antibiotic administration timing. The results indicated a significant difference in the timing of administration for multiple pregnancy, severe respiratory distress syndrome (RDS) symptoms, and the presence of an indwelling umbilical venous catheter (UVC) (*P* < 0.05). Twin pregnancy, more severe RDS symptoms, and UVC placement were identified as significant factors associated with the timing of antibiotic administration (*P* < 0.05), as shown in Table 1.

**Table 1:** The univariate factors associated with the time of initial antibiotic administration, assessed using the median and interquartile range (IQR)

Variables	Category	Time (h)	*P* value^*^
Multiple pregnancy	Singleton	3.6(3.0-4.9)	0.011
	Twin	5.0(3.2–6.4)	
Gestational age	< 28^+ 0^ weeks	4.8(3.2–5.2)	0.352
	28^+ 0^-32^+ 0^ weeks	3.7(3.0–5.0)	
Sex	Male	3.6(3.0–5.0)	0.359
	Female	4.3(3.2–5.1)	
Birth Weight	<1000	4.4(3.3–5.3)	0.671
	≥ 1000	3.7(3.1-5.0)	
Apgar score (5 min)	≥ 8	3.6(3.0-5.1)	0.452
	≤ 7	4.7(4.0-4.9)	
Mode of delivery	Vaginal	3.5(2.9-5.0)	0.152
	Emergency C-section	3.9(3.2–5.1)	
	Elective C-section	4.5(3.2–5.2)	
RDS grade	<II	3.4(3.0–5.0)	0.002
	≥II	4.5(3.2-6.0)	
Ventilation mode in the delivery room	Intubation	4.0(3.2-5.0)	0.848
	Noninvasive	3.7(3.0-5.1)	
Requirement for endotracheal intubation in the NICU	No	3.8(3.1-5.0)	0.940
	Yes	4.0(3.0-5.1)	
Surfactant administration in the NICU	No	4.0(3.2–5.1)	0.846
	Yes	3.6(3.0–5.0)	
Clinical signs of shock	No	3.9(3.2–5.1)	0.288
	Yes	3.0(2.8–4.7)	
Indwelling UVC	No	2.6(3.0-4.6)	0.025
	Yes	4.4(3.2–5.3)	

*The time variable exhibited a nonnormal distribution, and the P value was calculated using a t test after logarithmic transformation of the time variable

Notes: RDS: respiratory distress syndrome; UVC: umbilical venous catheter

### Correlation analysis

The findings of the correlation analysis indicated that there was no significant correlation between the timing of NICU bed allocation and the timing of antibiotic administration. However, a significant positive correlation was observed between the timing of the medical order and the timing of antibiotic administration, with a positive correlation coefficient (r) of 0.73 (*P* < 0.001). Likewise, we also identified a significant correlation between the timing of PST performance and antibiotic administration (*r* = 0.76, *P* < 0.001).

### Linear regression analysis

During the univariate analysis, several factors showed a significant association with the timing of antibiotic administration (*P* < 0.1). Notably, multiple pregnancy, RDS severity, indwelling UVC placement, the timing of medical orders, and PST postponement were identified as significant predictors of antibiotic administration timing.

To further investigate the independent factors associated with antibiotic administration timing, we conducted a linear regression analysis. The results indicated that RDS severity, medical order timing, and PST timing were independent factors, collectively explaining 60% of the variance (R square = 0.6). Higher RDS severity, PST postponement, and delayed medical orders were significantly positively correlated with delayed antibiotic administration (as detailed in Table 2).

**Table 2:** Linear regression analysis to investigate the independent factors affecting the time of antibiotic administration

Variables	β Estimate	95% CI for β	t	*P* value
Multiple pregnancy (Twin vs. Singleton)	0.05	-0.11, 0.01	1.55	0.126
RDS grade (≥II VS. <II)	0.07	0.12, 0.02	2.56	0.013
Indwelling UVC (Yes VS. No)	-0.01	-0.07, 0.04	-0.45	0.656
PST timing	0.06	0.03, 0.08	4.7	< 0.001
Medical order timing	0.04	0.01, 0.07	2.46	0.017

Abbreviations: UVC: umbilical venous catheter; PST: penicillin skin test

## Discussion

The results of this study indicate a notable delay in the initial administration of antibiotics for preterm newborns with suspected EOS in our NICU, with a median time of 3.8 h. Infants with greater RDS severity, PST postponement and delayed medical orders may experience delays in antibiotic administration.

EOS, with an incidence of 2–20% in developed countries, is a serious and potentially life-threatening condition in preterm newborns [1, 9]. In an adult study, delayed appropriate therapy in patients with Gram-negative bacteria infections, whether resistant or susceptible, is associated with a 70% increase in length of stay, a 65% increase in total in-hospital costs, and a 20% higher risk of in-hospital mortality or discharge to hospice [10]. The findings of our study suggest that there is a delay in the initial administration of antibiotics for preterm newborns with suspected EOS in our unit in China. Importantly, the delays we observed are consistent with the delays reported in other countries. In the study of infants who underwent an evaluation for sepsis in the NICU, the median time to administer antibiotics was 134 min in the initial month. Following dedicated educational and quality improvement interventions, there was a significant enhancement, reducing the median time to antibiotic administration to 58 min [11]. Therefore, the results of our study highlight the need for a multifaceted QI study on reducing delays in the administration of antibiotics, with a focus on timely administration to improve outcomes.

To address these delays and enhance the QI project, the investigation of influencing factors is essential. The findings of a previous study suggested that a multidisciplinary approach including rapid-response activation and pharmacist involvement can be effective in reducing delays in antibiotic administration [12]. In a study involving preterm infants with late-onset sepsis, the authors found that delays in antibiotic administration were associated with several factors, including delayed cerebrospinal fluid or urine sample collection, the absence of a peripheral intravenous catheter, and a longer time to fluid bolus administration [13]. Importantly, the causes of delayed antibiotic administration may differ among different countries depending on the specific situation. Our findings revealed several factors that were significantly associated with delayed antibiotic administration, including RDS severity, medical order timing, and PST timing.

Therefore, our findings highlight the need for interventions aimed at improving the timeliness of medical orders, particularly in the context of EOS in newborns. Such interventions might include targeted education for healthcare providers regarding the importance of timely medical orders and clear communication among team members to ensure that orders are placed promptly. The findings of this study also suggest that PST performance remains a challenge in NICUs in China because this procedure must be performed before penicillin usage, which is a main factor for the delay in antibiotic administration [8]. Similarly, RDS severity may also play a role in delays in antibiotic administration, potentially due to challenges in managing multiple aspects of care in critically ill neonates. These findings underscore the need for a multidisciplinary approach, such as performing the PST earlier and promoting close collaboration between clinicians and support staff.

To improve the timeliness of antibiotic administration in further QI projects, several strategies may be considered. First, the use of electronic health records and decision support tools may help to facilitate prompt ordering by doctors and processing of antibiotics, reducing the burden on doctors and nurses, particularly in cases where time is of the essence. Second, the PST is essential in most NICUs in China, and efforts should be made to perform the PST earlier after patient arrival. Finally, this may necessitate the development of a streamlined framework aimed at expediting medical order entry and establishing a support mechanism for nurses during periods of high workload.

## Conclusion

In conclusion, our study elucidates the pressing issue of delayed antibiotic administration in preterm newborns with suspected EOS in our unit. We found that several factors, including the severity of RDS, PST postponement and delayed medical orders, were significantly associated with delayed antibiotic administration in preterm infants with EOS.

### Limitations

Several limitations should be acknowledged regarding this study. First, as a single-center study, selection bias is possible, and the findings may not be generalizable to other settings. Second, since this investigation focused exclusively on preterm infants with a gestational age below 32 weeks, there may be limited applicability of the results to other populations. Third, we did not examine the influences of parental or caregiver factors, such as education level or socioeconomic status, which could impact the administration of antibiotics. Finally, while this study identified an association between delays in antibiotic administration and healthcare provider awareness, further research is needed to explore the underlying reasons for these delays, including system-level factors that may contribute to suboptimal care.

## Data Availability

The data supporting this study’s findings are available from the corresponding author upon request.
